# High-efficiency Rosa26 knock-in vector construction for Cre-regulated overexpression and RNAi

**DOI:** 10.1186/1755-8417-1-3

**Published:** 2008-11-03

**Authors:** Peter Hohenstein, Joan Slight, Derya Deniz Ozdemir, Sally F Burn, Rachel Berry, Nicholas D Hastie

**Affiliations:** 1 MRC Human Genetics Unit, Western General Hospital, Crewe Road, Edinburgh EH4 2XU, UK

## Abstract

**Introduction:**

Rosa26 is a genomic mouse locus commonly used to knock-in cDNA constructs for ubiquitous or conditional gene expression in transgenic mice. However, the vectors generally used to generate Rosa26 knock-in constructs show instability problems, which have a severe impact on the efficiency of the system.

**Results:**

We have optimized the cloning procedure to generate targeting vectors for Cre-regulated expression of constructs within several days with minimal hands-on time, thereby enabling high-throughput approaches. We demonstrate that transient expression of Cre still results in expression of the construct, as shown by the expression level and via functional assays. In addition to its well-established possibilities in expressing cDNA constructs, we show that the Rosa26 locus can be used to drive expression of functional miRNA constructs from its endogenous promoter.

**Conclusion:**

We provide a new high-efficiency cloning system for Rosa26 knock-in constructs to express either cDNA or miRNA fragments. Our system will enable high-throughput approaches for controlled expression of cDNA or miRNA constructs, with the latter providing a potential high-speed alternative for conditional knock-out models.

## Introduction

Over recent decades, genetically manipulated mouse models have proven themselves as valuable tools to study human disease, both as means to identify and validate new therapeutic intervention schemes and as models to study disease mechanisms. Broadly speaking, the genetics of human diseases can be divided into situations where the activity of a gene is ectopically activated or increased, or its endogenous activity is decreased or completely lost. Therefore, different types of mouse models mimicking these distinct situations are needed.

For ectopic expression of cDNA constructs in transgenic mice, the most commonly used method is zygotic pronuclear microinjection of complete expression cassettes consisting of enhancer and minimal promoter fragments, the cDNA of interest, a poly-A signal and sometimes an intron to enhance expression. However, the random integration of these constructs into the mouse genome causes much variability, due to copy number differences and position effects caused by the site of integration. As a result, different independent founder lines need to be tested and the usefulness of a model cannot be predicted. Even if a useful model to express a given cDNA in a desired tissue-specific pattern has been described, using the same regulatory elements does not guarantee that a different cDNA can be driven in a comparable manner. In addition, the random copy number of the integrated construct can result in artificially high expression levels. In some situations this is an advantage, but when attempting to mimic the situation found in human patients as closely as possible, this can be a severe problem.

An alternative approach is a knock-in of the cDNA of interest into a well-defined genomic locus using embryonic stem (ES) cell targetings followed by injection of correctly targeted ES cells into blastocysts, the generation of chimeric mice and germline transmission. The disadvantages of this approach are the more complex vector construction, the need for the identification of correctly targeted clones and the introduction of an extra generation of chimeric mice before the experimental mice are available. However, this is partially compensated for by a predictable expression pattern. More efficient ways to generate knock-in vectors for targeting into defined loci with high targeting efficiency would compensate for the loss in time resulting from the extra chimeric generation, if fewer independent mouse lines, or just a single line, would need to be analysed.

Conventional or conditional gene targeting are the most widely used methods of inactivating an endogenous gene. Whereas conventional targeting will only show the earliest (lethal) phenotype resulting from a mutation, conditional targeting allows tissue-specific and/or inducible inactivation of a gene into which loxP recombination sites have been introduced by controlling the expression of the Cre recombinase. This method can be very efficient, but the targeting vector can be complicated to make and targeting efficiencies can differ between loci. In addition, the effect of targeting an endogenous gene will in most cases be recessive, leading to additional breeding steps to generate the experimental mice.

In the last few years the use of siRNA in mammalian systems has been described as an *in vivo *alternative to targeting of endogenous genes [1], and Cre-regulated systems have been described for use in mouse models [2]. A major advantage of this technique is that expression of an RNAi construct is dominant, and would therefore reduce the breeding steps to generate experimental mice. A potential drawback is the fact that knock-down of a gene will generally not give a 100% loss of gene activity. However, as genetic aberrations found in patients are often hypomorphic mutations this could be considered an advantage, especially when attempting to mimic a human disease situation as closely as possible. In fact, an allelic series of different models for a given gene can provide valuable information on the role of a gene in a disease process [3]. Unfortunately, to achieve the efficiency needed for these types of experiments, most *in vivo *RNAi systems described make use of lentiviral vectors making them impractical for many laboratories.

The Rosa26 locus was originally identified in a gene-trap screen in murine ES cells, and was subsequently shown to be ubiquitously expressed during embryonic development and in adult mice [4,5]. The locus is believed to encode three non-coding transcripts of unknown function. In the original gene-trap line, the insertion of a splice acceptor site and promoter-less β-geo cDNA in intron 1 of the gene leads to coupling of its expression to the endogenous Rosa26 promoter. Although two of the three transcripts are disrupted, mice homozygous for this insertion are viable and apparently normal [5]. Subsequently it was shown that insertion of a lox-STOP-lox cassette between the splice acceptor site and cDNA places the expression of the cloned fragment under the control of Cre recombinase, as found in a commonly used transgenic Cre reporter mouse line [6]. Vectors designed to target the Rosa26 locus can be targeted with high efficiency (~25% is normal), making it a locus of choice for targeted knock-in mouse models.

Srinivas *et al *[7] described the pBigT vector, which provided a multiple cloning site and a two-step cloning procedure for Rosa26 knock-in vectors. Although potentially powerful, in practice its use can be severely complicated by an inherent instability of the Rosa26 backbone vector. In an attempt to streamline the generation of Cre-regulated Rosa26 targeting constructs we adapted the pBigT vector system to the Gateway cloning system, in which a combination of *att*-mediated *in vitro *recombination and strong negative counter-selection provides an efficient DNA shuttling system that can easily be used in high-throughput cloning projects [8]. Here we describe the use of this vector to generate both cDNA and miRNA constructs with minimal hands-on time.

## Results

### Generation and use of the pRosa26-DEST vector

**Figure 1:**
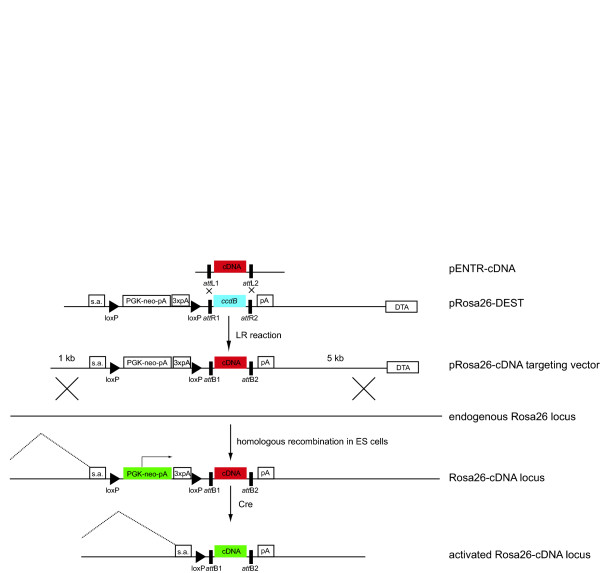
**Use of pRosa26-DEST for Cre-regulated expression of cDNA constructs**. Entry vectors carrying Gateway-compatible cDNA constructs without any regulatory sequences can be shuttled into pRosa26-DEST using a LR reaction in which the *ccdB *bacterial negative counter-selection gene is lost. The construct is recombined via gene targeting into the endogenous Rosa26 locus. The first exon of the Rosa26 locus (outside the targeting construct) will splice to the splice acceptor site in the construct but transcription is interrupted by the PGK-neo-pA and three copies of the SV40 polyA signal, which function as a STOP cassette. Cre-mediated removal of this cassette links Rosa26 exon 1 to the cDNA and thus the cDNA is expressed. As the endogenous Rosa26 transcripts are not translated into protein, the first start codon in the cDNA will be used for initiation of translation. Fragments shown in green can be expressed, fragments in red cannot; the *ccdB *counter-selection gene in blue is expressed in bacteria only.

To improve the efficiency with which Rosa26 knock-in vectors can be generated, we first adapted the pBigT vector [7] to the Gateway cloning system by inserting a Gateway entry cassette into the multiple cloning site of pBigT. Next, the splice acceptor-lox-STOP-lox-Gateway entry-poly-A cassette was subcloned into the Rosa26 backbone as for normal pBigT vectors to generate pRosa26-DEST. Using this vector, the insert from any Gateway-compatible Entry vector can be recombined into the final targeting vector in a single step (Figure 1). When generating knock-in constructs using pRosa26-DEST, we found it only took four days to generate the targeting vectors and have the DNA ready for targeting. These four days include the Gateway recombination reaction, transformation into *Escherichia coli*, testing several clones by miniprep and performing a maxiprep of a correct clone to isolate sufficient plasmid DNA for linearization and electroporation into ES cells. Most, if not all of these steps can easily be adapted to high-throughput approaches. In contrast to the pBigT cloning system, we have found that instability of the vector is not a limiting problem. We routinely find between 3 and 16 correct colonies when testing 16 *E. coli *colonies per construct (based on 10 cDNA constructs, data not shown). The efficiency of the cloning system has been confirmed in several collaborating laboratories (data not shown).

### Use of pRosa26-DEST for Cre-regulated expression of cDNA constructs

**Figure 2:**
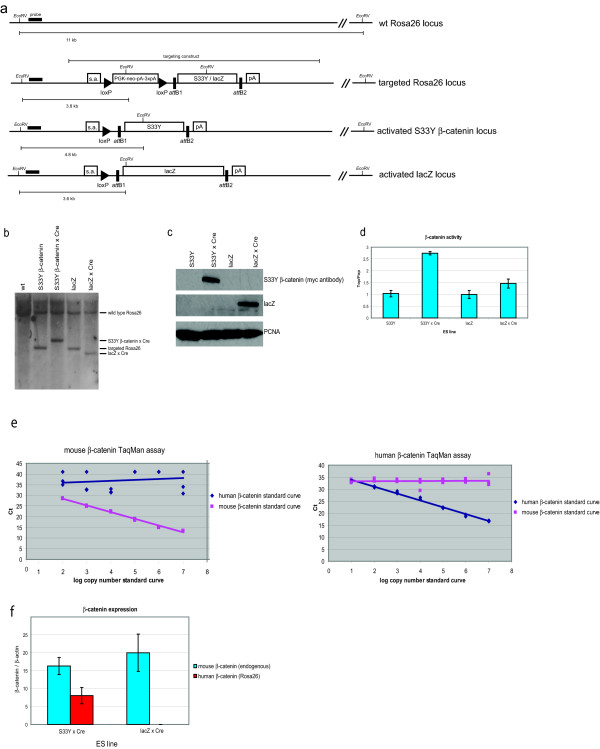
**pRosa26-DEST derived constructs are activated by expression of Cre**. (a) Screening scheme of pRosa26-DEST targeted embryonic stem (ES) clones via Southern blot. Note that the size of the detected fragment after activation of the construct with Cre depends on the exact position of an *Eco*RV site in the cDNA in the construct; if no *Eco*RV is present the 11 kb band found in wildtype cells will become longer. (b) Southern blot confirmation of correctly targeted and activated S33Y β-catenin and lacZ ES clones. (c) Western blot analysis of S33Y β-catenin and lacZ ES cells before and after activation of the construct using transient expression of Cre. (d) β-catenin activity measured as the ratio between TopFlash and FopFlash signals in S33Y β-catenin and lacZ ES cells. (e) Demonstration of specificity of the human and murine β-catenin TaqMan assays using absolute dilution curves of both amplification products. (f) Absolute quantification of β-catenin expression from endogenous locus (mouse β-catenin) and Rosa26 locus (human β-catenin).

As a proof of principle, we used pRosa26-DEST to express a mutant β-catenin cDNA under control of Cre. Activating mutations in the β-catenin proto-oncogene are found in a variety of human tumours and differences in β-catenin activity have been found to lead to different phenotypes [9], suggesting that physiologically relevant expression levels are important when modelling human diseases linked to β-catenin mutations in mice. We generated a Rosa26 knock-in vector carrying a *myc*-tagged S33Y mutant form of β-catenin, a patient-derived mutation that has been used in many *in vitro *studies. As a control we used pRosa26-DEST to knock-in lacZ into the Rosa26 locus (Figure 2a). We used transient transfection of a Cre expression plasmid to activate the expression of the S33Y β-catenin and lacZ cDNAs (Figure 2b). Western blot analysis showed that the introduction of the *att *recombination sequences in the construct does not disrupt correct expression regulation of the proteins (Figure 2c). To functionally test the Rosa26-DEST-derived constructs we measured β-catenin activity in the S33Y β-catenin and lacZ-expressing ES cells. As expected, the Super8xTOPFlash β-catenin reporter construct [10] showed an increased β-catenin transcriptional activity in ES cells expressing the S33Y mutation (Figure 2d). To test whether expression of β-catenin from the Rosa26 locus results in physiologically relevant expression levels, we quantified β-catenin expression with real-time reverse-transcriptase polymerase chain reaction (RT-PCR). As the mutant cDNA construct was based on the human cDNA, we could design mouse and human β-catenin specific TaqMan assays. We confirmed the specificity of the assays on a dilution series of both cDNA constructs (Figure 2e). Absolute quantification of mouse (endogenous locus) and human (Rosa26 locus) β-catenin showed that the expression level from the Rosa26 locus is approximately half that of the endogenous β-catenin locus (Figure 2f). As the endogenous β-catenin is present in two copies, while the Rosa26 knock-in messenger is expressed from a single copy, and in human cancer just one copy of β-catenin is activated by oncogenic mutations, we have achieved a highly relevant expression system for mutant β-catenin. As expected, the human β-catenin construct was not detected in the control Rosa26-lacZ cells. Several cDNA constructs generated with pRosa26-DEST, including the S33Y β-catenin allele, have now been passed through the germline and are currently being analysed, the results of which will be published elsewhere.

### Cre-regulated RNAi using pRosa26-DEST

**Figure 3:**
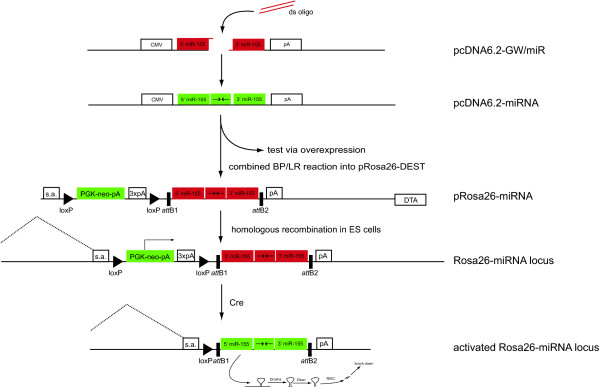
**Use of pRosa26-DEST for Cre-regulated RNAi**. Target sequences can be cloned as double-stranded oligonucleotides into a Gateway-compatible miRNA vector. This vector can directly be used to test different target sequences or the miRNA fragment can be transferred to pRosa26-DEST via a combined BP/LR reaction. The resulting vector is ready for targeting as described in Figure 1. Expression of the miRNA is blocked by the lox-STOP-lox cassette. Cre-mediated removal of this cassette links the miRNA to exon 1 of the endogenous Rosa26 locus to transcribe a chimeric transcript consisting of Rosa26 exon 1 and the miRNA sequence. The miRNA will be processed out of this primary transcript by Drosha, Dicer and the RISC complex and lead to knock-down of the target gene like other endogenous miRNAs.

**Figure 4:**
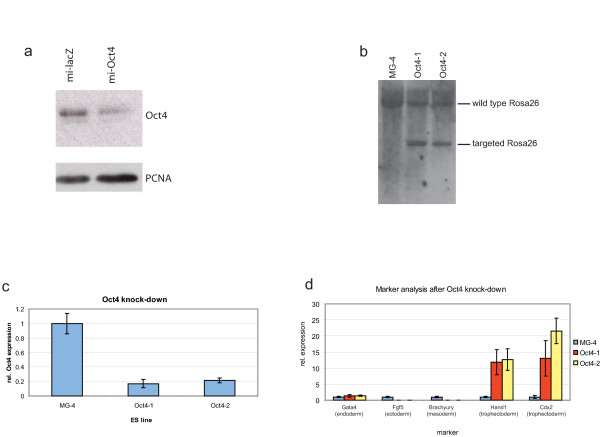
**Oct4 knock-down using pRosa26-DEST phenocopies the conventional knock-out phenotype**. (a) Western blot of undifferentiated embryonic stem (ES) cells transiently transfected with pcDNA6.2-GW/EmGFP-miR lacZ and pcDNA6.2-GW/EmGFP-miR Oct4. (b) Southern blot confirmation of two independent MG-4 derived ES clones targeted with the pRosa26-miOct4 construct. The screening strategy is the same as in Figure 2a before expression of Cre. (c) Oct4 expression after tamoxifen treatment to activate the CreERT2 in MG-4 cells and its derivates. (d) Marker analysis of Oct-4 knock-down ES cells.

In recent years RNAi has evolved into a powerful *in vitro *and *in vivo *technique to analyse gene function. By placing a siRNA target sequence of choice in the backbone of an endogenous miRNA, expression from RNA polymerase II promoters can be achieved [11,12], enabling the use of inducible and tissue-specific promoters in mice. Since endogenous miRNAs are processed out of larger transcripts [13], we reasoned that it should be possible to express artificial miRNAs from the Cre-regulated Rosa26 locus (Figure 3). We used a commercially available and Gateway-compatible miR-155-based vector and designed a miRNA construct against Oct4. It has been shown that this gene is essential for ES cells to maintain their undifferentiated state, and loss of Oct4 in ES cells in the presence of LIF results in specific differentiation towards the trophectoderm lineage [14], thereby providing a powerful functional test of the efficiency of RNAi using pRosa26-DEST. Using transient overexpression of the miRNA vector in undifferentiated ES cells (using the CMV promoter in the miRNA vector, see Figure 3), we confirmed the functionality of the target sequence (Figure 4a). Generation of a miRNA-expressing pRosa26-DEST construct was found to be as efficient as the generation of cDNA constructs. After subcloning of the miRNA fragment into pRosa26-DEST vector via a simple combined BP/LR reaction, we targeted the constructs to MG-4 ES cells, an E14-derived cell line that stably expresses a tamoxifen-regulatable Cre recombinase (Figure 4b). We activated expression of the miRNA using tamoxifen. In two independent Oct4 knock-down clones this resulted in the appearance of large flat, often multi-nucleated cells suggestive of trophectoderm (data not shown). Quantitative RT-PCR showed an approximately 80% reduction in Oct4 expression (see Figure 4c). Expression analysis of markers for different differentiation lineages confirmed that the cells were preferentially differentiated towards trophectoderm (see Figure 4d). These results were confirmed using a cell-permeable Cre to activate expression of the miRNA [15] (data not shown).

## Discussion

The Rosa26-DEST vector solves a major bottleneck in the generation of Rosa26 knock-in targeting vector construction. Its use is dependent on having the cDNA or miRNA in a Gateway-compatible vector. In our experience the most efficient way of cloning cDNA constructs is using a PCR and Gateway-mediated recombination approach. Using standard high-fidelity *Taq *polymerase and normal sequencing facilities we have generated several sequence verified clones in parallel, ready for recombination into pRosa26-DEST in four to five days, making the total time needed to generate constructs eight to nine days. At present we have generated and targeted 12 cDNA constructs which are in different stages of analysis in ES cells or mouse models.

By using the same vector for Cre-regulated RNAi we have described a high-speed system that provides a potential rapid alternative for normal conditional knock-out experiments. Using the method and vectors described here complete constructs can be made in only seven days with, again, minimal hands-on time. The specific differentiation towards trophectoderm as a result of Rosa26-mediated knock-out of Oct4 shows physiological-relevant phenotypes can be reproduced in ES cells. Its *in vivo *use needs to be confirmed, for which we are currently testing several constructs designed against disease genes where mutations in their human counterparts result in hypomorphic rather than complete loss-of-function alleles (data not shown). We believe that in many situations the use of Rosa26-DEST-based miRNA constructs can form a rapid alternative to conditional mouse models, while different target sequences against the same gene can easily provide allelic series when gene dosage effects are to be studied [3]. The possibility of linking different miRNA targets in the same construct [12] should enable parallel knock-down of different genes in the same mouse without the need for time-consuming breeding schemes.

Use of the Rosa26 or any other endogenous locus is likely to result in lower expression levels than found in conventional transgenics, as these will have higher copy numbers and often use artificial promoters selected for high expression. Our real-time RT-PCR data shows that expression from the Rosa26 locus results in physiological relevant expression levels, and might therefore be preferable when the purpose of the model is to accurately mimic the situation in a human disease. In fact, use of vectors such as pRosa26-DEST might help dissect gene-specific phenotypes from phenotypes (partially) caused by overexpression artefacts. If the highest possible expression level is needed, then the Multisite Gateway system should provide the possibility of including custom promoter elements, selected for high, tissue-specific or inducible expression in pRosa26-DEST-based constructs. This approach might also be useful for the docking of reporter constructs into the Rosa26 locus. Preliminary experiments show these types of constructs can indeed be made and several constructs are currently being tested (data not shown). Another alternative to achieve high expression of Cre-regulatable transgenes is the CLIP vector [16]. This vector has the additional advantage of providing positive selection for Cre-mediated activation of the expression cassette in ES cells; however, its random integration into the genome has the potential to inactivate endogenous genes.

We are currently testing different ways to further improve the targeting efficiency in order to obtain to a system where we no longer need to test single ES clones for correct targeting, but can instead rely on using pools of stable, neomycin-resistant clones. Combined with the development of cell-permeable Cre proteins [15] for testing constructs in ES cells and the recently described methods for generation of fully ES-derived embryos and mice from commonly used inbred ES lines [17], we envision a 'gene analysis pipeline' that will greatly accelerate the *in vivo *functional annotation of the mouse genome.

## Conclusion

The pRosa26-DEST vector provides an efficient system to generate Cre-regulated knock-in constructs for the Rosa26 locus. It can be used for expression of cDNA and miRNA constructs. As such, it can provide a rapid alternative for the generation of mouse models with predictable expression patterns and levels.

## Methods

### Vector construction

All oligos were from Invitrogen (standard purification). pBigT-DEST was generated by digesting pBigT [7] with *Xho*I and blunted using Klenow polymerase. The C1 cassette from the Gateway Conversion System (Invitrogen) was inserted and transformed into DB3.1 bacteria (Invitrogen) and selected for combined Amp/Cam resistance. Restriction digests were used to check the orientation of the Gateway cassette. To generate pRosa26-DEST the *Pac*I/*Asc*I fragment from pBigT-DEST was transferred to pRosa26/PA [7]. pBigT and pRosa26/PA were a kind gift from Dr Frank Costantini (Columbia University, New York). The pRosa26-DEST vector was checked for unwanted recombination events using PCRs for different parts of the vector and sequencing of essential regions.

S33Y β-catenin was amplified using bcat-attB/F-myc (GGGACAAGTTTGTACAAAAAAGCAGGCTTCACCATGGAACAAAAACTCATCTCAGAAGAGGATCTGATGGCTACTCAAGCTGATTTG) and bcat-attB/R (GGGGACCACTTTGTACAAGAAAGCTGGGTCCTACAGGTCAGTATCAAACCAGG). The forward primer contains an *att*B1 site followed by a Kozak sequence, *myc *epitope tag and 20 nt homology to the 5' end of the human β-catenin cDNA. The reverse primer contained 20 nt homology to the 3' end of human β-catenin, stop codon and *att*B2 sequences. A cDNA construct with S33Y human β-catenin (a kind gift from Dr Marc van de Wetering, Hubrecht Laboratory, Utrecht) was used as a template. The fragment was amplified using the Expand High Fidelity PCR system (Roche) and cloned into pDONR-221 using BP clonase enzyme mix (Invitrogen). PCR inserts were sequence verified in both orientations. The resulting vector was designated pENTR-S33 β-catenin. To generate pENTR-lacZ, the lacZ coding region from pLenti4/TO/V5-GW/lacZ was transferred to pDONR-221 using a BP reaction. For the final targeting vectors pRosa26-DEST was mixed with pENTR-S33 β-catenin or pENTR-lacZ in a 6-hour LR recombination reaction using LR clonase enzyme mix (Invitrogen), followed by transformation into Stbl3 *E. coli *cells and grown at 30°C. For each construct 16 clones were miniprepped and several correct clones were identified for each based on *Pac*I/*Asc*I digest and *Kpn*I digest.

miRNA targets against Oct4 were designed using the BLOCK-iT RNAi designer [18]. Oligos Oct4-846-TOP (TGCTGTACAGAACCATACTCGAACCAGTTTTGGCCACTGACTGACTGGTTCGAATGGTTCTGTA) and Oct4-846-BOT (CCTGTACAGAACCATTCGAACCAGTCAGTCAGTGGCCAAAACTGGTTCGAGTATGGTTCTGTAC) were cloned into pcDNA6.2-GW/EmGFP-miR (Invitrogen) according to the supplier's instructions. The supplied miLacZ oligos were used to generate a control miRNA construct against lacZ. Inserted oligos were sequence verified using the supplied sequencing primers. The Oct4 miRNA vector was mixed with pDONR-221 for a 6-hour BP reaction, immediately followed by an overnight LR reaction with pRosa26-DEST as recipient vector and transformed into Stbl3 cells. Again several correct clones could be identified based on *Pac*I/*Asc*I and *Kpn*I digestions of 16 miniprepped clones. The resulting construct was designated pRosa26-miOct4-846.

All targeting vectors were maxiprepped using Qiagen Plasmid Maxi kit and 100 μg vector was linearized with *Kpn*I. Digestions were ethanol precipitated overnight at -20°C, spun down, air-dried in a cell culture flow cabinet, dissolved in phosphate buffered saline (PBS) and used for electroporation of ES cells.

### ES cell culture

E14IVtg2a cells (10^7^) were grown on feeder-free gelatinized plates in LIF-supplemented medium and electroporated with 100 μg linearized (*Kpn*I) targeting vector and selected for 9 to 10 days in G418 (250 μg/ml). Single clones were picked, expanded, frozen and tested for correct targeting using Southern blot as described previously [7].

MG-4 cells were derived from E14IVtg2a cells by electroporating them with 100 μg of a CAGGs-CreERT2-IRES-puro construct (a kind gift from Dr Lars Grotewold, ISCR, University of Edinburgh) and selection on puromycin (Invivogen, final concentration 1 μg/ml). Several independent puromycin-resistant clones were tested and shown to express CreERT2 using Western blot analysis. One clone, MG-4, was shown to give germline transmission after generation of chimeric mice. MG-4 cells were electroporated with the pRosa26-miOct4-846 construct as described above.

4-OH Tamoxifen (Sigma) was dissolved in ethanol to 1 mg/ml (1000 × final concentration) and stored at -20°C. For activation of Cre in MG-4-derived cells, 7.5 × 10^6 ^cells were grown overnight in T75 flasks in normal ES medium. The next day 4-OH Tamoxifen or ethanol for controls was added and cells were grown for an additional three days with daily medium changes. Finally the cells were grown for another three days in normal ES medium without 4-OH Tamoxifen or ethanol.

### Western blot analysis

Lysates were made using Complete Lysis-M (Roche) and 50 μg was run on sodium dodecyl sulfate polyacrylamide gel electrophoresis (SDS-PAGE) minigels. Antibodies were 9E10 (Santa Cruz, recognizing the *myc *epitope on S33Y β-catenin), C10 (Santa Cruz sc-5279, recognizing Oct3/4), CR7001RPZ (Cortex, recognizing lacZ) and AB407 (Autogen Bioclear, recognizing PCNA as the loading control).

### β-catenin activity assay

8xSuperTOPFlash and 8xSuperFOPFlash (kind gifts from Dr Randall Moon, Howard Hughes Medical Institute, University of Washington) [10] were used to measure β-catenin activity. ES cells were plated in 96 well plates at a density of 12500 cells per well and grown overnight. Cells were transfected overnight with 62.5 ng 8xTOPFlash or 8xFOPFlash construct, 62.5 ng pRL-TK (Promega) and 0.3125 μl Lipofectamine-2000 (Invitrogen) per well. Luciferase was measured using the Dual-Luciferase Reporter Assay System (Promega).

### Real-time PCR

RNA was isolated using the RNeasy mini kit (Qiagen) with DNAse treatment on the column. Five micrograms total RNA was transcribed with AMV (Avian Myeloblastosis Virus) reverse transcriptase (Roche) and oligo(dT) priming. All quantitative PCR assays were performed on an ABI 7900 HT real-time PCR system with FastStart TaqMan Master (Rox) (Roche). Assays were performed with the Universal Probe Library (Roche) with the primer/probe combinations shown in Table 1. Cdx2 was measured against a standard curve of cDNA derived from embryoid bodies grown for 10 days; Oct4, Gata4, Fgf5, Brachyury and Hand1 were measured against a standard curve from embryoid bodies grown for six days. For absolute quantification cDNA fragments containing the real-time PCR fragments of β-actin, human β-catenin and murine β-catenin were cloned in pGemT-easy (Promega), sequence verified, diluted to exact copy numbers and used as standard curve.

**Table 1:** Primers and UPL probes used in the real-time reverse-transcriptase polymerase chain reaction experiments

**Transcript**	**Forward**	**Reverse**	**Probe**
**Oct4**	GTTGGAGAAGGTGGAACCAA	CTCCTTCTGCAGGGCTTTC	95
**β-actin**	AAGGCCAACCGTGAAAAGAT	GTGGTACGACCAGAGGCATAC	56
**Gata4**	GGAAGACACCCCAATCTCG	CATGGCCCCACAATTGAC	13
**Fgf5**	AAAACCTGGTGCACCCTAGA	CATCACATTCCCGAATTAAGC	29
**Brachyury**	CGACCACAAAGATGTAATGGAG	CCAGCACCAGGAACAAGC	66
**Hand1**	AGCGGAAAAGGGAGTTGC	GCGCCCTTTAATCCTCTTCT	51
**Cdx2**	CTGCCACACTTGGGCTCT	CTTGGCTCTGCGGTTCTG	34
**Human β-catenin**	GCAGAGTGCTGAAGGTGCTA	TCTGTCAGGTGAAGTCCTAAAGC	31
**Mouse β-catenin**	ATGAAGGCGTGGCAACATAC	TGTGGCTTGTCCTCAGACAT	56

A VectorNTI archive file (additional file 1), sequence (additional file 2), map (additional file 3) of pRosa26-DEST and detailed protocols (additional file 4) are available as supplementary information to this paper. For reagent requests, please see [19].

## Competing interests

The authors declare that they have no competing interests.

## Authors' contributions

PH designed the study, generated constructs, performed ES experiments and wrote the manuscript. JS performed ES experiments and protein analysis. DDO generated constructs and performed real-time PCR. SFB and RB generated constructs. NDH assisted in the design of the study and helped in drafting the manuscript. All authors read and approved the final manuscript.

## Supplementary Material

Additional file 1 VectorNTI archive file for pRoas26-DEST. Click here for file

Additional file 2 pRosa26-DEST sequence in genbank format. Click here for file

Additional file 3 pRosa26-DEST map. Click here for file

Additional file 4 pRosa26-DEST information file including protocols. Click here for file
