# Supplementary figures and images for: High-efficiency Rosa26 knock-in vector construction for Cre-regulated overexpression and RNAi
Source: Pathogenetics. 2008 Nov 3;1:3. doi: 10.1186/1755-8417-1-3 (PMC2583990; doi:10.1186/1755-8417-1-3)

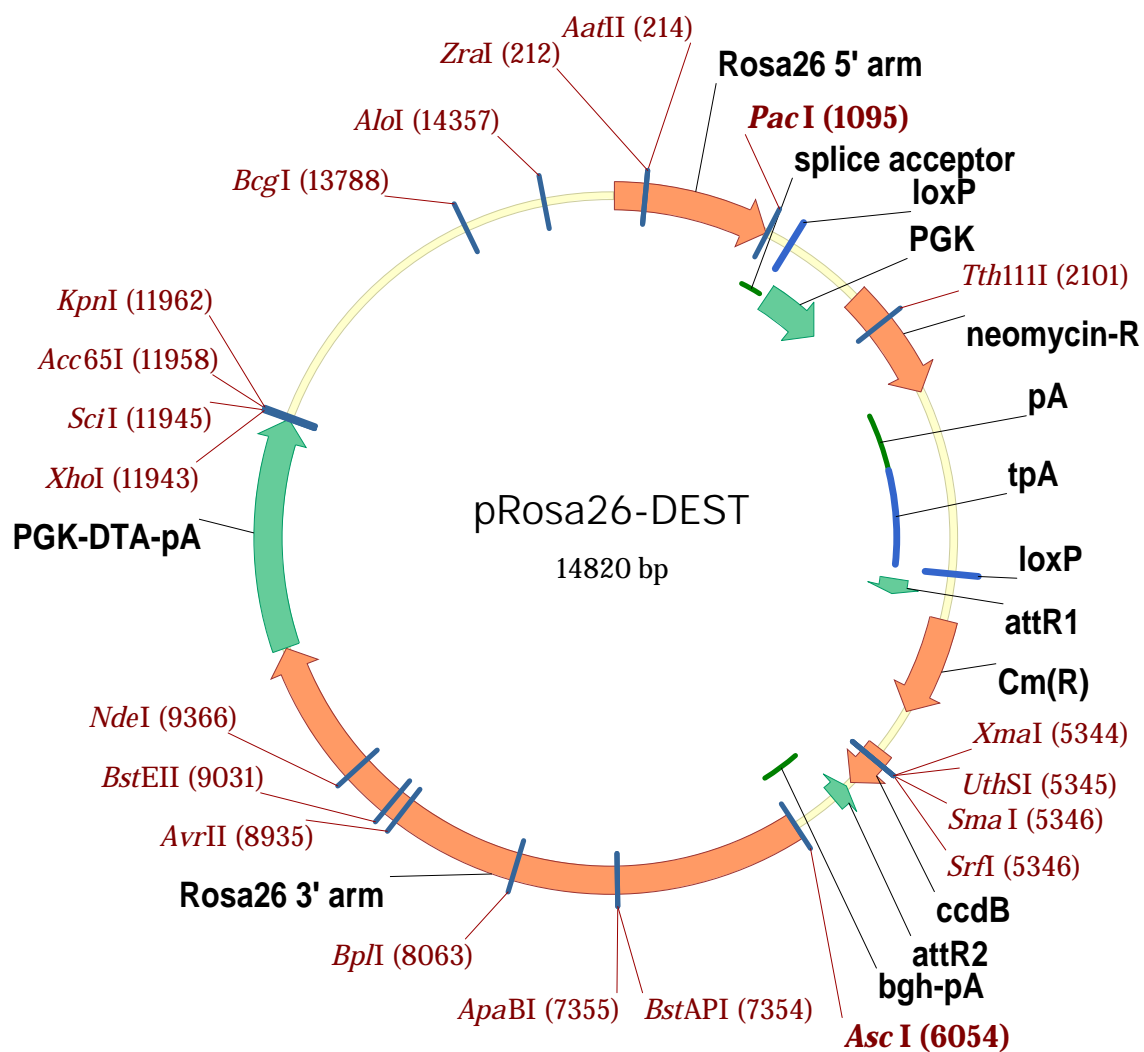

Supplement: Additional file 3 — pRosa26-DEST map. [file 1755-8417-1-3-S3.pdf]
