# Supplementary material for: High-efficiency Rosa26 knock-in vector construction for Cre-regulated overexpression and RNAi
Source: Pathogenetics. 2008 Nov 3;1:3. doi: 10.1186/1755-8417-1-3 (PMC2583990; doi:10.1186/1755-8417-1-3)
Supplement: Additional file 4 — pRosa26-DEST information file including protocols. [file 1755-8417-1-3-S4.pdf]

Vector construction with pRosa26-DEST

Peter Hohenstein

This overview is based on my experience with the GateWay system and the pRosa26-DEST vector in particular. Everything (vector and information) comes ‘as it is’ without any warranties. Feel free to contact me with questions, tips, problems, ideas or if you want to be kept informed on further improvements of the vector at **peter.hohenstein@hgu.mrc.ac.uk**

pRosa26-DEST was made by inserting a GateWay conversion cassette into the *Xho*I site of pBigT, followed by transfer of *Pac*I / *Asc*I fragment of the resulting vector to the pRosa26-PA vector (see **http://www.biomedcentral.com/1471-213X/1/4** for details on these vectors). The resulting vector resembles the construct that was used for the R26R Cre reporter mouse made by Phil Soriano in which the expression of lacZ is coupled to the endogenous Rosa26 locus via a splice acceptor site, but only after removal of the lox-STOP-lox cassette (which is actually the neo-R cassette used during the targeting). The only thing changed from the pBigT vectors and the R26R reporter is the cloning strategy, so the resulting knock-in should be as good as these systems, and as good (or as bad) as the use of the Rosa26 locus in any situation.

<u>Generating Entry vectors for pRosa26 knock-in vectors</u>

More detailed information on this is available in the GateWay manual (http://www.invitrogen.com/content/sfs/manuals/gatewayman.pdf). Please also check this for general information on the GateWay system.

Briefly, the options are as follows:

1. Use conventional cloning to insert your cDNA of interest in one of the pENTR vectors (http://www.invitrogen.com/content/sfs/manuals/pentr_man.pdf). This can be straightforward when you're lucky with restriction sites. However, it is important to check reading frame and unexpected start / stop codons.
2. Generate a PCR fragment with primers extended with *att*B sites and recombine into a pDONR vector (http://www.invitrogen.com/content/sfs/manuals/pdonr_man.pdf) using a BP reaction. This is my favourite method, since it gives complete control over start and stop signals and it allows extra sequences like epitope or purification tags to be included. Disadvantage is the sequencing of several clones that will be needed to check for PCR errors.
3. TOPO clone a PCR fragment without *att* sites added to the primers into an appropriate vector (http://www.invitrogen.com/downloads/B-12645_Gateway_TOPO.pdf) . Will still need to be sequenced.
4. If you have a GateWay expression construct with your cDNA of interest, you can perform a BP reaction on this vector with a pDONR vector and grow bacteria on kanamycin selection. The resulting clones will be Entry clones with your cDNA.
5. You can buy sequence verified GateWay Entry clones from Invitrogen and several not-for-profit clone distribution sources.

You can check reading frame etc. of your construct at the design stage using Vector NTI, which is these days freely available for not-for-profit researchers (https://catalog.invitrogen.com/index.cfm?fuseaction=userGroup.home). With this program you can generate your Entry vector *in silico*, perform an *in silico* LR reaction with a DEST vector that has a CMV promoter and analyse the ORF in the resulting expression vector before proceeding to spending bench time and money. However, you can also do this with old-fashioned pen, paper and common sense.

Generating the Rosa26 knock-in vectors for cDNA expression

pRosa26-DEST contains the lethal *ccdB* counter selection gene used in the GateWay system. Therefore it can only be maintained in bacteria that are insensitive to this gene, like DB3.1 cells. The supplied bacteria are from this strain and carry the vector. If you need to retransform the vector, you will need to use DB3.1 cells.
The original pBigT vector system (http://www.srinivas.org/) on which pRosa26-DEST is based is notorious for its instability. In pRosa26-DEST this problem is partially but not completely solved. My experience so far with generating 10 different constructs shows that at least 3 / 16 *E.coli* clones tested are correct. For this I standard use Stbl3 cells, which carry an extra mutation that makes instable constructs like lentivirus vectors more stable. I also grow the cells at 30° C to be on the safe side. I never tried standard DH5α cells at 37° C.

I use the pRosa26-DEST with maxi-prep quality; my Entry vectors are usually mini-prep, in some cases I used mini-prep from an automated robotics system with some of the magnetic beads still in it and it worked fine, so apparently quality of this DNA is not the most critical factor.

1. Perform a standard 10 μl LR reaction:
    150 ng pRosa26-DEST
    150 ng Entry vector
    TE to 8 μl
    2 μl LR clonase II

2. Incubate 4-6 h at rt.

3. Add 1 μl ProtK (2 μg/μl, supplied with LR enzyme mix) and incubate 10' at 37° C. I often actually take 5μl in a fresh tube and treat this with ProtK, while incubating the remainder o/n at rt (just in case, I never actually needed this).

4. Transform 2-3 μl into One-Shot Stbl3 cells according to the supplied protocol. I let them recover shaking for 1 ½ h rather then 1 h at 30° C.

5. Plate on Amp plates and grow o/n at 30° C.

6. The next day pick 16 colonies and grow o/n at 30° for miniprep

7. On day 3 I first test all minipreps by digesting with *Kpn*I (or it isoschizomer *Asp*718), which will linearize the vector, any unwanted recombination will give a clearly shorter fragment than you would expect. The clones that look correct on this digest I test further with *Pac*I/*Asc*I double digest (NEB buffer 4 + BSA) which will release the fragment as it was originally derived from pBigT, which will be easier to check the length after inserting your cDNA (remember that the GateWay reaction not only removes the sequence between the *att* sites, but that the *att*R sites from pRosa26-DEST will be replaced by *att*B sites which are only 25 nt each instead of 125, which will reduce the length of the fragment with 200 nt). I run the samples on 0.6-0.7% TAE gels at a relatively low voltage (max. 80 V).

8. Grow a 400 ml maxiprep of a correct clone o/n at 30° C.

9. Maxiprep the vector, take up DNA in 150 μl TE and measure concentration.

10. Test 1 μl DNA again with *Kpn*I and *Pac*I/*Asc*I and if correct I digest 100 μg vector with *Kpn*I for electroporation (if your cDNA contains a *Kpn*I site, you should also be able to use *Xho*I, *Sci*I, *Acc*65I, *Bcg*I or *Alo*I for linearization, though I never had to test this).

11. After digestions and testing 1 μl on gel for proper digestion, precipitate the DNA and keep it o/n at -20° C. The tube is ready to take into the ES lab.

<u>Generating the Rosa26 knock-in vectors for Cre regulated RNAi</u>

The idea of using the Rosa26 system for Cre-regulated RNAi is based on the fact that endogenous miRNAs are processed out of bigger RNA polymerase II transcribed transcripts (from introns or UTRs) by the Drosha complex, before further processing by Dicer and RISC into functional siRNAs. Since the Rosa26 knock-in systems will generate a fusion transcript between exon 1 of the Rosa26 transcript and the inserted sequence, it would recapitulate a normal situation for the expression of miRNA molecules. The work of the labs of Brian Cullen, Greg Hannon and others has shown that replacing the target sequence of an endogenous miRNA by a target sequence against your favourite gene will result in efficient knockdown of this gene (better than the comparable shRNA, especially at low copy numbers, likely due to better processing by Drosha and Dicer) from RNA polymerase II driven constructs and with better target sequence design rules.

The protocol described here is based in the Gateway-compatible miRNA vectors from Invitrogen pcDNA6.2-GW/miR (http://www.invitrogen.com/content/sfs/vectors/pcdna6_2gw_miR_map.pdf) and pcDNA6.2-GW/EmGFP-miR (http://www.invitrogen.com/content/sfs/vectors/pcdna6_2gw_miR_map.pdf). These vectors are based on the mouse miR-155 miRNA and target sequences are easily cloned into the system as dsDNA 64-mer oligos. I have generated another Gateway-compatible miRNA vector based on the human miR-30 backbone as used in the Hannon shRNA^{mir} library and using their cloning method. Though I'm sure their shotgun cloning and sequence-till-you-drop method is great for their whole genome libraries, I didn't find the method efficient enough (at least in my hands) for just few specific constructs at a time. Two other advantages of the Invitrogen vectors are the possibilities of linking GFP expression to the expression of the miRNA and linking several miRNA sequences in the same construct (either against the same gene to increase knockdown further if needed or against different genes to knockdown more then one gene with one construct). So far I haven't seen the GFP expression when targets where knocked into the Rosa locus (though I haven't looked too hard yet, in overexpression situations it's working great). My guess is that at low copy numbers (as in the single copy Rosa26 knock-in) all expressed transcripts are processed by Drosha, leading to loss of the GFP mRNA. In contrast, when doing overexpression experiments you're saturating the miRNA processing machinery, so the GFP you'll see is basically the leftovers. I've no hard data to support this, but in overexpression experiment we've seen GFP-negative cells that have great knockdown (I guess these are cells with only a few copies of the vector) but never GFP-positive cells that didn't show the knockdown. I haven't tried the linking of target sequence yet, but I cannot think of a reason why this wouldn't work when knocked into the Rosa26 locus.

Biggest disadvantage of the Invitrogen vectors is the fact that they're being sold as lineralized vectors that you cannot maintain yourself. Personally I think that the speed of making constructs with these vectors more than compensates for this, but if people want to try my miR-30 based vector just contact me. Note that I haven't used these in the Rosa locus yet.

1. Design the 2 64-mer oligos using the design tool on the Invitrogen website (https://rnaidesigner.invitrogen.com/rnaiexpress/). After hybridising them the dsDNA oligo will have the correct overhang to insert into the Invitrogen vectors.
2. Order the cheapest quality oligos that you think you can get away with, in my experience this means the cheapest oligos from either Invitrogen or Sigma are fine, don't bother even trying cheap Eurogentec oligos.
3. Dilute and hybridise the oligos as described in the manual that comes with the vector.
4. After hybridising I continue directly with the ligation as described in the manual, I never test the oligos on gel. I usually ligate for 1h but I have done 5 min, which gave fewer colonies but still more than enough.
5. After ligation proceed immediately with transformation, when plating don't forget the vectors are Spec resistant, not Amp or Kan.
6. I sometimes get a mixture of small and large colonies. If this is the case, pick the large ones and grow o/n for miniprep. I usually grow 4 colonies / construct
7. Have the minipreps sequenced to check for mutations in the oligos. Sequencing the hairpins can be tricky, in our hands we get good results using 30 pmol of the forward sequencing

primer as described in the vector manual per reaction with the addition of 5% DMSO to the sequencing reaction.

8. When correct clones are identified, they can directly be used for overexpression experiments (for instance for testing). At this stage the GFP cassette can be removed if needed, or different target sequences can be linked, for all this see the vector manual. Alternatively, the miRNA (with or without GFP) can be shuttled into pRosa26-DEST in a combined LR/BP reaction.
9. Perform a BP reaction:
   150 ng pDONR221 (circular)
   150 ng miRNA vector (circular)
   TE to 8 µl
   2 µl BP clonase II

10. Incubate 6 h at rt
11. Perform a LR reaction:
    3 µl BP reaction
    150 ng pRosa26-DEST (circular)
    TE to 8 µl
    2 µl LR clonase II

12. Incubate for o/n at rt
13. Add 1 µl ProtK and incubate 10’ at 37° C
14. Briefly cool reaction on ice
15. Transform 3 µl to Stbl3 cells
16. Plate on Amp-R plates and grow o/n at 30° C
17. Check colonies via miniprep and digestion as described for the cDNA constructs.

Targeting with pRosa26-DEST based vectors

All our experiments so far have been with E14-IV cells, which are 129Ola. If you're using cells from another cell line or genetic background I'd be interested in your results.

We routinely electroporate $1x10^7$ cells with 100 µg linearized vector. Cells are plated on 10 x 100 mm dishes and grown o/n without selection. The next day we put our cells on neomycin selection. We pick colonies after 10 days selection. The vector targets with approximately 25% efficiency, so on average we pick 48-60 clones. This way we can always choose between sufficient numbers of good looking clones.

Clones are tested via Southern blot. The probe can be isolated from pRosa-5' (see http://www.fhcrc.org/science/labs/soriano/vectors/pROSA26-5prime.html) with *Eco*RI/*Hin*dIII). The probe gives a wt band of 11 kb and a correctly targeted band of 3.8 kb on a *Eco*RV digest of genomic DNA. It doesn't give nice blots, but good enough for scoring.

We're currently using a first PCR screen to test colonies for correct homologues recombination. Based on these results we select a few clones to expand, freeze and confirm on Southern.
The PCR is based on the protocol as found on Phil Soriano's website. The most important part of the protocol is the PCR buffer. I've spend months and months being too lazy to make this buffer and just trying new primer sets with normal PCR buffer, without any success. With this buffer the PCR works like a treat. The primer sequences given here are different from the primers on Phil Soriano's website. They were designed and tested by Wenhao Xu (University of Virginia) and work better in our hands than the other primers. Finally, a hot start is important, but since we're doing our PCRs in 96 well plates with adhesive lids I don't find a normal hot start very convenient. The Invitrogen Platinum taq is an automatic hot start taq that works great in this PCR without any hassle.

Rosa26 PCR screen of ES clones

1. After picking, ES clones are grown in 96 well plates
2. Continue growing until medium in 80% of the wells turn orange-yellow in one day (change medium on all wells daily once the first wells turn yellow)
3. Split plate in 2 new gelatinised plates: plate A gets 20% of the cells, plate B gets 80%.
4. Next day wash cells in plate B with PBS and put 200 µl lysis buffer on cells
5. Grow o/n at 37° C
6. Next day use 1 µl lysate in a 25 µl PCR reaction and analyse on 1.2% agarose gel. I usually continue with 4 PCR-positive clones.
7. Continue growing cells in plate A until wells are 70-80% confluent
8. Trypsinize selected wells and transfer complete well to 24 wells well
9. When 70-80% confluent passage complete well to 5 new wells on 24 wells plate
10. Grow to 80% confluency, pool cells from 4 wells and freeze in 8 vials for injection and *in vitro* activation of the construct, grow 5th well completely confluent for DNA isolation for Southern blot confirmation

Lysis buffer (12 ml):

10 x GB buffer:	1.2 ml
10% Triton X-100:	0.6 ml
milliQ H_2O:	10.2 ml
store at 4° C	

before use add 0.4 µl ProtK (20 mg/ml) / 200 µl lysis buffer; I dissolve ProtK in 50% glycerol for storage at –20° C to prevent subsequent freezing/thawing cycles)

10 x GB buffer (10 ml):

2M TRIS pH 8.8:	3.35 ml
1 M $(NH_4)_2SO_4$:	1.66 ml
0.5 M $MgCl_2$:	1.34 ml
milliQ H_2O:	3.65 ml

store at -20° C

PCR mix (1 sample):

10 x MGB buffer:	2.5 µl
10 x dNTP (2 mM each):	2.5 µl
DMSO:	2.5 µl
F primer (12,5 µM):	0.2 µl
R primer (12,5 µM):	0.2 µl
Invitrogen Platinum Taq:	0.2 µl
β-mercapto-ethanol:	0.125 µl
milliQ H_2O:	11.775 µl

10 x MGB buffer (10 ml):

2M TRIS pH 8.8:	3.35 ml
1 M $(NH_4)_2SO_4$:	1.66 ml
0.5 M $MgCl_2$:	1.30 ml
0.5% gelatine:	2.00 ml
milliQ H_2O:	1.69 ml

PCR conditions:

3’ 95° C
45 X 20” 94° C
30” 62° C
2’30” 68° C
7’ 72° C
10’ 4° C
end

primer sequences:
F: GGCGGACTGGCGGGACTA
R: GGGACAGGATAAGTATGACATCATCAAGG

Fragment length approx. 1.5 kb

Some handy cat. no. for Invitrogen stuff you might need…

BP clonase II: 11789020
LR Clonase II: 11791020
Stbl3 cells: C7373-03
DB3.1 cells: 11782-018

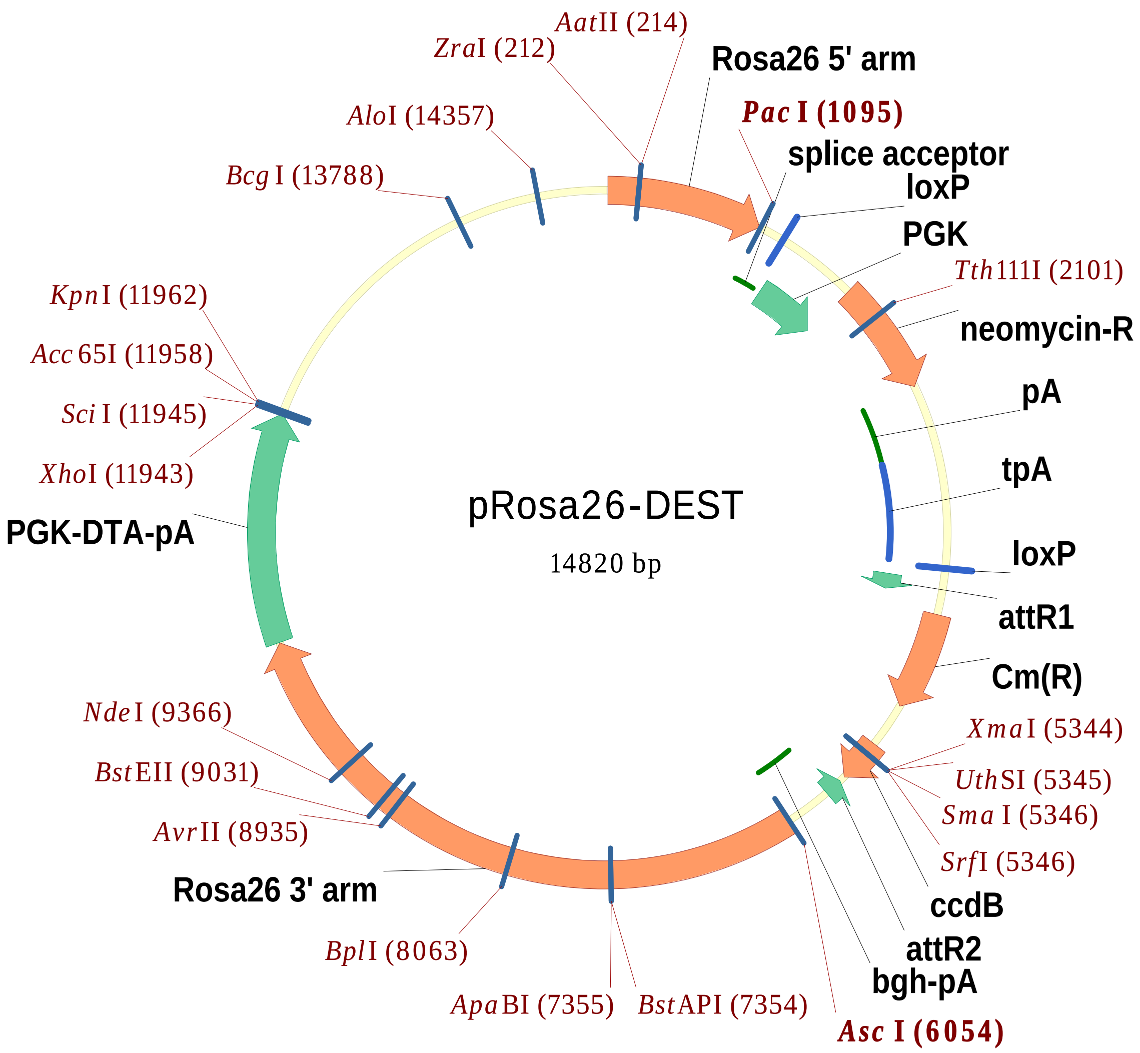

pRosa26-DEST
14820 bp
AatII (214)
ZraI (212)
AloI (14357)
BcgI (13788)
Rosa26 5' arm
PacI (1095)
splice acceptor
loxP
PGK
Tth111I (2101)
neomycin-R
pA
tpA
loxP
attR1
Cm(R)
XmaI (5344)
UthSI (5345)
SmaI (5346)
SrfI (5346)
ccdB
attR2
bgh-pA
AscI (6054)
BstAPI (7354)
ApaBI (7355)
BplI (8063)
Rosa26 3' arm
AvrII (8935)
BstEII (9031)
NdeI (9366)
PGK-DTA-pA
XhoI (11943)
SciI (11945)
Acc65I (11958)
KpnI (11962)

```
LOCUS       pRosa26-DEST          14820 bp    DNA     circular     9-MAR-2006
DEFINITION  GateWay destination vector for knock-in in Rosa26 locus.
SOURCE
  ORGANISM
COMMENT     This file is created by Vector NTI
            http://www.invitrogen.com/
COMMENT     VNTDATE|404447100|
COMMENT     VNTDBDATE|404447684|
COMMENT     LSOWNER|
COMMENT     VNTNAME|pRosa26-DEST|
COMMENT     VNTAUTHORNAME|Peter Hohenstein|
COMMENT     VNTREPLTYPE|Plasmid
COMMENT     VNTEXTCHREPL|Bacteria
FEATURES             Location/Qualifiers
     loci            1..1084
                     /vntifkey="18"
                     /label=Rosa26\5'\arm
     loci            6065..10335
                     /vntifkey="18"
                     /label=Rosa26\3'\arm
     misc_feature    10337..11945
                     /vntifkey="21"
                     /label=PGK-DTA-pA
     polyA_signal    5738..6051
                     /vntifkey="25"
                     /label=bgh-pA
     splicing_signal 1098..1271
                     /vntifkey="38"
                     /label=splice\acceptor
     misc_feature    1272..1305
                     /vntifkey="21"
                     /label=loxP
     promoter        1329..1840
                     /vntifkey="29"
                     /label=PGK
     exon            1845..2648
                     /vntifkey="61"
                     /label=neomycin-R
     polyA_signal    2649..3123
                     /vntifkey="25"
                     /label=pA
     terminator      3124..3909
                     /vntifkey="43"
                     /label=tpA
     misc_feature    3938..3971
                     /vntifkey="21"
                     /label=loxP
     CDS             5262..5567
                     /vntifkey="4"
                     /label=ccdB
     misc_recomb     complement(5608..5732)
                     /vntifkey="86"
                     /label=attR2
     CDS             4262..4942
                     /vntifkey="4"
                     /label=Cm(R)
     misc_recomb     4029..4153
                     /vntifkey="86"
                     /label=attR1
BASE COUNT     3648 a     3385 c     3681 g     4088 t      18 others
```

ORIGIN

```
        1 ccccgcggca ggccctccga gcgtggtgga gccgttctgt gagacagccg ggtacgagtc
       61 gtgacgctgg aaggggcaag cgggtggtgg gcaggaatgc ggtccgccct gcagcaaccg
      121 gagggggagg gagaagggag cggaaaagtc tccaccggac gcggccatgg ctcggggggg
      181 ggggggcagc ggaggascgc ttccggccga cgtctcgtcg ctgattggct tyttttcctc
      241 ccgccgtgtg tgaaaacaca aatggcgtgt tttggttggc gtaaggcgcc tgtcagttaa
      301 cggcagccgg agtgcgcagc cgccggcagc ctcgctctgc ccactgggtg ggcgggagg
      361 taggtggggt gaggcgagct gnacgtgcgg gcgcggtcgg cctctggcgg ggcgggggag
      421 gggagggagg gtcagcgaaa gtagctcgcg cgcgagcggc cgcccaccct ccccttcctc
      481 tgggggagtc gttttacccg ccgccggccg ggcctcgtcg tctgattggc tctcggggcc
      541 cagaaaactg gcccttgcca ttggctcgtg ttcgtgcaag ttgagtccat ccgccggcca
      601 gcgggggcgg cgaggaggcg ctcccaggtt ccggccctcc cctcggcccc gcgccgcaga
      661 gtctggccgc gcgcccctgc gcaacgtggc aggaagcgcg cgctgggggc ggggacgggc
      721 agtagggctg agcggctgcg gggcgggtgc aagcacgttt ccgacttgag ttgcctcaag
      781 aggggcgtgc tgagccagac ctccatcgcg cactccgggg agtggaggga aggagcgagg
      841 gctcagttgg gctgttttgg aggcaggaag cacttgctct cccaaagtcg ctctgagttg
      901 ttatcagtaa gggagctgca gtggagtagg cggggagaag gccgcaccct tctccggagg
      961 ggggagggga gtgttgcaat acctttctgg gagttctctg ctgcctcctg gcttctgagg
     1021 accgccctgg gcctgggaga atcccttgcc ccctcttccc ctcgtgatct gcaactccag
     1081 tctttctagt taattaaggg atctgtaggg cgcagtagtc cagggtttcc ttgatgatgt
     1141 catacttatc ctgtcccttt tttttccaca gctcgcggtt gaggacaaac tcttcgcggt
     1201 ctttccagtg gggatcgacg gtatcgtaga gtcgaggccg ctctagaact agtggatccg
     1261 gaacccttaa tataacttcg tataatgtat gctatacgaa gttattaggt ccctcgacct
     1321 gcaggaattc taccgggtag gggaggcgct tttcccaagg cagtctggag catgcgcttt
     1381 agcagccccg ctggcacttg gcgctacaca agtggcctct ggcctcgcac acattccaca
     1441 tccaccggta gcgccaaccg gctccgttct ttggtggccc cttcgcgcca ccttctactc
     1501 ctcccctagt caggaagttc ccccccgccc cgcagctcgc gtcgtgcagg acgtgacaaa
     1561 tggaagtagc acgtctcact agtctcgtgc agatggacag caccgctgag caatggaagc
     1621 gggtaggcct ttggggcagc ggccaatagc agctttgctc cttcgctttc tgggctcaga
     1681 ggctgggaag gggtgggtcc gggggcgggc tcaggggcgg gctcaggggc ggggcgggcg
     1741 cgaaggtcct cccgaggccc ggcattctcg cacgcttcaa aagcgcacgt ctgccgcgct
     1801 gttctcctct tcctcatctc cgggcctttc gacctgcagc caatatggga tcggccattg
     1861 aacaagatgg attgcacgca ggttctccgg ccgcttgggt ggagaggcta ttcggctatg
     1921 actgggcaca acagacaatc ggctgctctg atgccgccgt gttccggctg tcagcgcagg
     1981 ggcgcccggt tctttttgtc aagaccgacc tgtccggtgc cctgaatgaa ctgcaggacg
     2041 aggcagcgcg gctatcgtgg ctggccacga cgggcgttcc ttgcgcagct gtgctcgacg
     2101 ttgtcactga agcgggaagg gactggctgc tattgggcga agtgccgggg caggatctcc
     2161 tgtcatctca ccttgctcct gccgagaaag tatccatcat ggctgatgca atgcggcggc
     2221 tgcatacgct tgatccggct acctgcccat tcgaccacca agcgaaacat cgcatcgagc
     2281 gagcacgtac tcggatggaa gccggtcttg tcgatcagga tgatctggac gaagagcatc
     2341 aggggctcgc gccagccgaa ctgttcgcca ggctcaaggc gcgcatgccc gacggcgatg
     2401 atctcgtcgt gacccatggc gatgcctgct tgccgaatat catggtggaa aatggccgct
     2461 tttctggatt catcgactgt ggccggctgg gtgtggcgga ccgctatcag gacatagcgt
     2521 tggctacccg tgatattgct gaagagcttg gcggcgaatg ggctgaccgc ttcctcgtgc
     2581 tttacggtat cgccgctccc gattcgcagc gcatcgcctt ctatcgcctt cttgacgagt
     2641 tcttctgagg ggatccgctg taagtctgca gaaattgatg atctattaaa caataaagat
     2701 gtccactaaa atggaagttt ttcctgtcat actttgttaa gaagggtgag aacagagtac
     2761 ctacattttg aatggaagga ttggagctac gggggtgggg gtggggtggg attagataaa
     2821 tgcctgctct ttactgaagg ctctttacta ttgctttatg ataatgtttc atagttggat
     2881 atcataattt aaacaagcaa aaccaaatta agggccagct cattcctccc actcatgatc
     2941 tatagatcta tagatctctc gtgggatcat tgtttttctc ttgattccca ctttgtggtt
     3001 ctaagtactg tggtttccaa atgtgtcagt ttcatagcct gaagaacgag atcagcagcc
     3061 tctgttccac atacacttca ttctcagtat tgttttgcca agttctaatt ccatcagaag
     3121 cttgcagatc tgcgactcta gaggatctgc gactctagag gatcataatc agccatacca
     3181 catttgtaga ggttttactt gctttaaaaa acctcccaca cctccccctg aacctgaaac
     3241 ataaaatgaa tgcaattgtt gttgttaact tgtttattgc agcttataat ggttacaaat
     3301 aaagcaatag catcacaaat ttcacaaata aagcattttt ttcactgcat tctagttgtg
     3361 gtttgtccaa actcatcaat gtatcttatc atgtctggat ctgcgactct agaggatcat
     3421 aatcagccat accacatttg tagaggtttt acttgcttta aaaaacctcc cacacctccc
     3481 cctgaacctg aaacataaaa tgaatgcaat tgttgttgtt aacttgttta ttgcagctta
     3541 taatggttac aaataaagca atagcatcac aaatttcaca aataaagcat ttttttcact
     3601 gcattctagt tgtggtttgt ccaaactcat caatgtatct tatcatgtct ggatctgcga
     3661 ctctagagga tcataatcag ccataccaca tttgtagagg ttttacttgc tttaaaaaac
     3721 ctcccacacc tccccctgaa cctgaaacat aaaatgaatg caattgttgt tgttaacttg
     3781 tttattgcag cttataatgg ttacaaataa agcaatagca tcacaaattt cacaaataaa
     3841 gcattttttt cactgcattc tagttgtggt ttgtccaaac tcatcaatgt atcttatcat
     3901 gtctggatcc ccatcaagct gatccggaac ccttaatata acttcgtata atgtatgcta
     3961 tacgaagtta ttaggtccct cgacctgcag cccaagctag cttatcgata ccgtcgacct
```

4021 cgaatcaaac aagtttgtac aaaaaagctg aacgagaaac gtaaaatgat ataaatatca
4081 atatattaaa ttagattttg cataaaaaac agactacata atactgtaaa acacaacata
4141 tccagtccta tgggcggccg cattaggcac cccaggcttt acactttatg cttccggctc
4201 gtataatgtg tggattttga gttaggatcc gtcgagattt tcaggagcta aggaagctaa
4261 aatggagaaa aaaatcactg gatataccac cgttgatata tcccaatggc atcgtaaaga
4321 acattttgag gcatttcagt cagttgctca atgtacctat aaccagaccg ttcagctgga
4381 tattacggcc tttttaaaga ccgtaaagaa aaataagcac aagttttatc cggcctttat
4441 tcacattctt gcccgcctga tgaatgctca tccggaattc cgtatggcaa tgaaagacgg
4501 tgagctggtg atatgggata gtgttcaccc ttgttacacc gttttccatg agcaaactga
4561 aacgttttca tcgctctgga gtgaatacca cgacgatttc cggcagtttc tacacatata
4621 ttcgcaagat gtggcgtgtt acggtgaaaa cctggcctat ttccctaaag ggtttattga
4681 gaatatgttt ttcgtctcag ccaatccctg ggtgagtttc accagttttg atttaaacgt
4741 ggccaatatg gacaacttct tcgcccccgt tttcaccatg ggcaaatatt atacgcaagg
4801 cgacaaggtg ctgatgccgc tggcgattca ggttcatcat gccgtttgtg atggcttcca
4861 tgtcggcaga atgcttaatg aattacaaca gtactgcgat gagtggcagg gcggggcgtaa
4921 tctagaggat ccggcttact aaaagccaga taacagtatg cgtatttgcg cgctgatttt
4981 tgcggtataa gaatatatac tgatatgtat acccgaagta tgtcaaaaag aggtatgcta
5041 tgaagcagcg tattacagtg acagttgaca gcgacagcta tcagttgctc aaggcatata
5101 tgatgtcaat atctccggtc tggtaagcac aaccatgcag aatgaagccc gtcgtctgcg
5161 tgccgaacgc tggaaagcgg aaaatcagga agggatggct gaggtcgccc ggtttattga
5221 aatgaacggc tcttttgctg acgagaacag ggctggtga aatgcagttt aaggtttaca
5281 cctataaaag agagagccgt tatcgtctgt ttgtggatgt acagagtgat attattgaca
5341 cgcccgggcg acggatggtg atccccctgg ccagtgcacg tctgctgtca gataaagtcc
5401 cccgtgaact ttacccggtg gtgcatatcg gggatgaaag ctggcgcatg atgaccaccg
5461 atatggccag tgtgccggtc tccgttatcg gggaagaagt ggctgatctc agccaccgcg
5521 aaaatgacat caaaaacgcc attaacctga tgttctgggg aatataaatg tcaggctccc
5581 ttatacacag ccagtctgca ggtcgaccat agtgactgga tatgttgtgt tttacagtat
5641 tatgtagtct gtttttatg caaaatctaa tttaatatat tgatatttat atcattttac
5701 gtttctcgtt cagctttctt gtacaaagtg gttcgattcg aggggccccc gcgggcggcc
5761 gcgagctcgc tgatcagcct cgactgtgcc ttctagttgc cagccatctg ttgtttgccc
5821 ctcccccgtg ccttccttga ccctggaagg tgccactccc actgtccttt cctaataaaa
5881 tgaggaaatt gcatcgcatt gtctgagtag gtgtcattct attctggggg gtggggtggg
5941 gcaggacagc aagggggagg attgggaaga caatagcagg catgctgggg atgcggtggg
6001 ctctatggct tctgaggcgg aaagaaccag ctggggctcg atcctctagt tggcgcgccc
6061 tagaagatgg gcgggagtct tctgggcagg cttaaaggct aacctggtgt gtgggcgttg
6121 tcctgcaggg gaattgaaca ggtgtaaaat tggagggaca agacttccca cagattttcg
6181 gttttgtcgg gaagtttttt aataggggca aataggaaaa tggaggatag gagtcatctg
6241 gggtttatgc agcaaaacta caggtatatt gcttgtatcc gcctcggaga tttccatgag
6301 gagataaaga catgtcaccc gagtttatac tctcctgctt agatcctact acagtatgaa
6361 atacagtgty gcgaggtaga ctatgtaagc agatttaatc attttaaaga gcccagtact
6421 tcatatccat ttctcccgct ccttctgcag ccttatcaaa aggtatttag aacactcatt
6481 ttagccccat tttcatttat tatactggct tatccaaccc ctagacagag cattggcatt
6541 ttccctttcc tgatcttaga agtctgatga ctcatgaaac cagacagatt agttacatac
6601 accacaaatc gaggctgtag ctggggcctc aacactgcag ttcttttata actccttagt
6661 acacttttttg ttgatccttt gccttgatcc ttaattttca gtgtctatca cctctcccgt
6721 caggtggtgt tccacatttg ggcctattct cagtccaggg agttttacaa caatagatgt
6781 attgagaatc caacctaaag cttaactttc cactcccatg aatgcctctc tccttttttct
6841 ccattataac tgagctatwa ccattaatgg tttcaggtgg atgtctcctc ccccaatata
6901 cctgatgtat ctacatattg ccaggctgat attttaagac atwaaaggta tatttcatta
6961 ttgagccaca tggtattgat tactgctact aaaattttgt cattgtacac atctgtaaaa
7021 ggtggttcct tttggaatgc aaagttcagg tgtttgttgt ctttcctgac ctaaggtctt
7081 gtgagcttgt atttttttcta tttaagcagt gctttctctt ggactggctt gactcatggc
7141 attctacacg ttattgctgg tctaaatgtg attttgccaa gcttcttcag gacctataat
7201 tttgcttgac ttgtagccaa acacaagtaa aatgattaag caacaaatgt atttgtgaag
7261 cttggttttt aggttgttgt gttgtgtgtg cttgtgctct ataataatac tatccagggg
7321 ctggagaggt ggctcggagt tcaagagcac agactgctct tccagaagtc ctgagttcaa
7381 ttcccagcaa ccacatggtg gctcacaacc atctgtaatg ggatctgatg ccctcttctg
7441 gtgtgtctga agaccacaag tgtattcaca ttaaataaat aatcctcctt cttcttcttt
7501 tttttttttt aaagagaatw ctgtctccag tagaattact gaagtaatga aatactttgt
7561 gtttgttcca atatggwagc caataatcaa atactcttwa gcactggaaa tgtaccaagg
7621 aactatttta tttaagtgwa ctgtggacag aggagccata actgcagact tgtgggatac
7681 agaagaccaa tgcagactta atgtcttttc tcttacacta agcaataaag aaataaaaat
7741 tgaacttcta gtatcctatt tgttaaactg ctagctttac taacttttgt gcttcatcta
7801 tacaaagctg aaagctaagt ctgcagccat tactaaacat gaaagcaagt aatgataatt
7861 ttggatttca aaaatgtagg gccagagttt agccagccag tggtggtgct tgcctttatg
7921 ccttaatccc agcactctgg aggcagagac aggcagatct ctgagtttga gcccagcctg
7981 gtctacacat caagttctat ctaggatagc caggaataca cacagaaacc ctgttgggga
8041 gggggctct gagatttcat aaaattataa ttgaagcatt ccctaatgag ccactatgga

8101 tgtggctaaa tccgtctacc tttctgatga gatttgggta ttattttttc tgtctctgct
8161 gttggttggg tcttttgaca ctgtgggctt tcttaaagcc tccttccctg ccatgtggtc
8221 tcttgtttgc tactaacttc ccatggctta aatggcatgg ctttttgcct tctaagggca
8281 gctgctgagw tttgcagcct gatttccagg gtggggttgg gaaatctttc aaacactaaa
8341 attgtccttt aatttttttt taaaaaatgg gttatataat aaacctcata aaatagttat
8401 gaggagtgag gtggactaat attaatgagt ccctccccta taaaagagct attaaggctt
8461 tttgtcttat actaactttt tttttaaatg tggtatcttt agaaccaagg gtcttagagt
8521 tttagtatac agaaactgtt gcatcgctta atcagatttt ctagtttcaa atccagagaa
8581 tccaaattct tcacagccaa agtcaaatta agaatttctg actttaatgt tatttgctac
8641 tgtgaatata aaatgatagc ttttcctgag gcagggtctc actatgtatc tctgcctgat
8701 ctgcaacaag atatgtagac taaagttctg cctgcttttg tctcctgaat actaaggtta
8761 aaatgtagta atacttttgg aacttgcagg tcagattctt ttatagggga cacactaagg
8821 gagcttgggt gatagttggt aaatgtgttt aagtgatgaa aacttgaatt attatcaccg
8881 caacctactt tttaaaaaaa aaagccaggc ctgttagagc atgctaaggg atccctagga
8941 cttgctgagc acacaagagt agtacttggc aggctcctgg tgagagcata tttcaaaaaa
9001 caaggcagac aaccaagaaa ctacagtaag gttacctgtc tttaaccatc tgcatataca
9061 cagggatatt aaaatattcc aaataatatt tcattcaagt tttcccccat caaattggga
9121 catggatttc tccggtgaat aggcagagtt ggaaactaaa caaatgttgg ttttgtgatt
9181 tgtgaaattg ttttcaagtg atagttaaag cccatgagat acagaacaaa gctgctattt
9241 cgaggtctct tggttatact cagaagcact tctttgggtt tccctgcact atcctgatca
9301 tgtgctaggc ctwccttagg ctgattgttg ttcaaataac ttaagtttcc tgtcaggtga
9361 tgtcatatga tttcatatat caaggcaaaa catgttatat atgttaaaca tttgkactta
9421 atgtgaaagt taggtctttg tgggttttga ttttaatttc aaaacctgag ctaaataagt
9481 cattttacat gtcttacatt tggtgaattg tatattgtgg tttgcaggca agactctctg
9541 acctagtaac cctcctatag agcactttgc tgggtcacaa gtctaggagt caagcatttc
9601 accttgaagt tgagacgttt tgttagtgta tactagttat atgttggagg acatgtttat
9661 ccagaagata ttcaggacta tttttgactg ggctaaggaa ttgattctga ttagcactgt
9721 tagtgagcat tgagtggcct ttaggcttga attggagtca cttgtatatc tcaaataatg
9781 ctggcctttt ttwaaaagcc cttgttcttt atcaccctgt tttctacata atttttgttc
9841 aaagaaatac ttgtttggat ctccttttga caacaatagc atgttttcaa gccatatttt
9901 ttttcctttt tttttttttt tttggttttt cgagacaggg tttctctgta tagccctggc
9961 tgtcctggaa ctcactttgt agaccaggct ggcctcgaac tcagaaatcc gcctgcctct
10021 gcctcctgag tgccgggatt aaaggcgtgc accaccacgc ctggctaagt tggatatttt
10081 gtatataact ataaccaata ctaactccac tgggtggatt tttaattcag tcagtagtct
10141 taagtggtct ttattggccc ttattaaaat ctactgttca ctctaacaga ggctgttgga
10201 ctagtggsac taagcaactt cctacggata tactagcaga taagggtcag ggatagaaac
10261 tagtctagcg ttttgtatac ctaccagctt atactacctt gttctgatag aaatatttag
10321 gacatctagc ttatcgatcc gtcgacggta tcgataagct tgatatcgaa ttctaccggg
10381 taggggaggc gcttttccaa ggcagtctga gcatgcgctt agcagccccg ctggcacttg
10441 gcgctacaca agtggcctyt ggcctcgcac acattccaca tccaccggta ggcgccaacc
10501 ggctccgttc tttggtggcc ccttcgcgcc accttctwct cctcccctag tcaggaagtt
10561 cccccccgcc ccgcagctcg cgtcgtsagg acgtgacaaa tggaagtagc acgtctcact
10621 agtctcgtca gatggacagc accgctgagc aatggaagcg ggtaggcctt tggggcagcg
10681 gccaatagca gctttgctcc ttcgctttct gggctcagag gctgggaagg ggtgggtccg
10741 ggggcgggct caggggcggg ctcaggggcg gggcgggcgc ccgaaggtcc tccggaggcc
10801 cggcattctg cacgcttcaa aagcgcacgt ctgccgcgct gttctcctct tcctcatctc
10861 cgggcctttc gacctgcagg tcctcgccat ggatcctgat gatgttgtta ttcttctaat
10921 cttttgtatg gaaaactttt cttcgtacca cgggactaaa cctggttatg tagattccat
10981 tcaaaaaggt atacaaaagc caaaatctgg tacacaagga aattatgacg atgattggaa
11041 agggttttat agtaccgaca ataaatacga cgctgcggga tactctgtag ataatgaaaa
11101 cccgctctct ggaaaagctg gaggcgtggt caaagtgacg tatccaggac tgacgaaggt
11161 tctcgcacta aaagtggata atgccgaaac tattaagaaa gagttaggtt taagtctcac
11221 tgaaccgttg atggagcaag tcggaacgga agagtttatc aaaaggttcg gtgatggtgc
11281 ttcgcgtgta gtgctcagcc ttcccttcgc tgaggggagt tctagcgttg aatatattaa
11341 taactgggaa caggcgaaag cgttaagcgt agaacttgag attaattttg aaacccgtgg
11401 aaaacgtggc caagatgcga tgtatgagta tatggctcaa gcctgtgcag gaaatcgtgt
11461 caggcgatct ctttgtgaag gaaccttact tctgtggtgt gacataattg gacaaactac
11521 ctacagagat ttaaagctct aaggtaaata taaaattttt aagtgtataa tgtgttaaac
11581 tactgattct aattgtttgt gtattttaga ttccaaccta tggaactgat gaatgggagc
11641 agtggtggaa tgcagatcct agagctcgct gatcagcctc gactgtgcct tctagttgcc
11701 agccatctgt tgtttgcccc tcccccgtgc cttccttgac cctggaaggt gccactccca
11761 ctgtcctttc ctaataaaat gaggaaattg catcgcattg tctgagtagg tgtcattcta
11821 ttctgggggg tggggtgggg caggacagca agggggagga ttgggaagac aatagcaggc
11881 atgctgggga tgcggtgggc tctatggctt ctgaggcgga aagaaccagc tggggctcga
11941 cctcgagggg gggcccggta cccagctttt gttcccttta gtgagggtta attgcgcgct
12001 tggcgtaatc atggtcatag ctgtttcctg tgtgaaattg ttatccgctc acaattccac
12061 acaacatacg agccggaagc ataaagtgta aagcctgggg tgcctaatga gtgagctaac
12121 tcacattaat tgcgttgcgc tcactgcccg ctttccagtc gggaaacctg tcgtgccagc

12181 tgcattaatg aatcggccaa cgcgcgggga gaggcggttt gcgtattggg cgctcttccg
12241 cttcctcgct cactgactcg ctgcgctcgg tcgttcggct gcggcgagcg gtatcagctc
12301 actcaaaggc ggtaatacgg ttatccacag aatcagggga taacgcagga aagaacatgt
12361 gagcaaaagg ccagcaaaag gccaggaacc gtaaaaaggc cgcgttgctg gcgtttttcc
12421 ataggctccg cccccctgac gagcatcaca aaaatcgacg ctcaagtcag aggtggcgaa
12481 acccgacagg actataaaga taccaggcgt ttccccctgg aagctccctc gtgcgctctc
12541 ctgttccgac cctgccgctt accggatacc tgtccgcctt tctcccttcg ggaagcgtgg
12601 cgctttctca tagctcacgc tgtaggtatc tcagttcggt gtaggtcgtt cgctccaagc
12661 tgggctgtgt gcacgaaccc cccgttcagc ccgaccgctg cgccttatcc ggtaactatc
12721 gtcttgagtc caacccggta agacacgact tatcgccact ggcagcagcc actggtaaca
12781 ggattagcag agcgaggtat gtaggcggtg ctacagagtt cttgaagtgg tggcctaact
12841 acggctacac tagaaggaca gtatttggta tctgcgctct gctgaagcca gttaccttcg
12901 gaaaaagagt tggtagctct tgatccggca aacaaaccac cgctggtagc ggtggttttt
12961 ttgtttgcaa gcagcagatt acgcgcagaa aaaaaggatc tcaagaagat cctttgatct
13021 tttctacggg gtctgacgct cagtggaacg aaaactcacg ttaagggatt ttggtcatga
13081 gattatcaaa aaggatcttc acctagatcc ttttaaatta aaaatgaagt tttaaatcaa
13141 tctaaagtat atatgagtaa acttggtctg acagttacca atgcttaatc agtgaggcac
13201 ctatctcagc gatctgtcta tttcgttcat ccatagttgc ctgactcccc gtcgtgtaga
13261 taactacgat acgggagggc ttaccatctg gccccagtgc tgcaatgata ccgcgagacc
13321 cacgctcacc ggctccagat ttatcagcaa taaaccagcc agccggaagg gccgagcgca
13381 gaagtggtcc tgcaacttta tccgcctcca tccagtctat taattgttgc cgggaagcta
13441 gagtaagtag ttcgccagtt aatagtttgc gcaacgttgt tgccattgct acaggcatcg
13501 tggtgtcacg ctcgtcgttt ggtatggctt cattcagctc cggttcccaa cgatcaaggc
13561 gagttacatg atcccccatg ttgtgcaaaa aagcggttag ctccttcggt cctccgatcg
13621 ttgtcagaag taagttggcc gcagtgttat cactcatggt tatggcagca ctgcataatt
13681 ctcttactgt catgccatcc gtaagatgct tttctgtgac tggtgagtac tcaaccaagt
13741 cattctgaga atagtgtatg cggcgaccga gttgctcttg cccggcgtca atacgggata
13801 ataccgcgcc acatagcaga actttaaaag tgctcatcat tggaaaacgt tcttcggggc
13861 gaaaactctc aaggatctta ccgctgttga gatccagttc gatgtaaccc actcgtgcac
13921 ccaactgatc ttcagcatct tttactttca ccagcgtttc tgggtgagca aaaacaggaa
13981 ggcaaaatgc cgcaaaaaag ggaataaggg cgacacggaa atgttgaata ctcatactct
14041 tcctttttca atattattga agcatttatc agggttattg tctcatgagc ggatacatat
14101 ttgaatgtat ttagaaaaat aaacaaatag gggttccgcg cacatttccc cgaaaagtgc
14161 cacctaaatt gtaagcgtta atattttgtt aaaattcgcg ttaaattttt gttaaatcag
14221 ctcatttttt aaccaatagg ccgaaatcgg caaaatccct tataaatcaa aagaatagac
14281 cgagataggg ttgagtgttg ttccagtttg gaacaagagt ccactattaa agaacgtgga
14341 ctccaacgtc aaagggcgaa aaaccgtcta tcagggcgat ggcccactac gtgaaccatc
14401 accctaatca agttttttgg ggtcgaggtg ccgtaaagca ctaaatcgga accctaaagg
14461 gagcccccga tttagagctt gacggggaaa gccggcgaac gtggcgagaa aggaagggaa
14521 gaaagcgaaa ggagcgggcg ctagggcgct ggcaagtgta gcggtcacgc tgcgcgtaac
14581 caccacaccc gccgcgctta atgcgccgct acagggcgcg tcccattcgc cattcaggct
14641 gcgcaactgt tgggaagggc gatcggtgcg ggcctcttcg ctattacgcc agctggcgaa
14701 agggggatgt gctgcaaggc gattaagttg ggtaacgcca gggttttccc agtcacgacg
14761 ttgtaaaacg acggccagtg agcgcgcgta atacgactca ctatagggcg aattggagct
//
